# Supplementary material for: Real-Life Vancomycin Therapeutic Drug Monitoring in Coagulase-Negative Staphylococcal Bacteremia in Neonatal and Pediatric Intensive Care Unit: Are We Underestimating Augmented Renal Clearance?
Source: Antibiotics (Basel). 2023 Oct 26;12(11):1566. doi: 10.3390/antibiotics12111566 (PMC10668724; doi:10.3390/antibiotics12111566)
Supplement: Supplementary file 1 [file antibiotics-12-01566-s001.zip › antibiotics-2611315-supplementary.pdf]

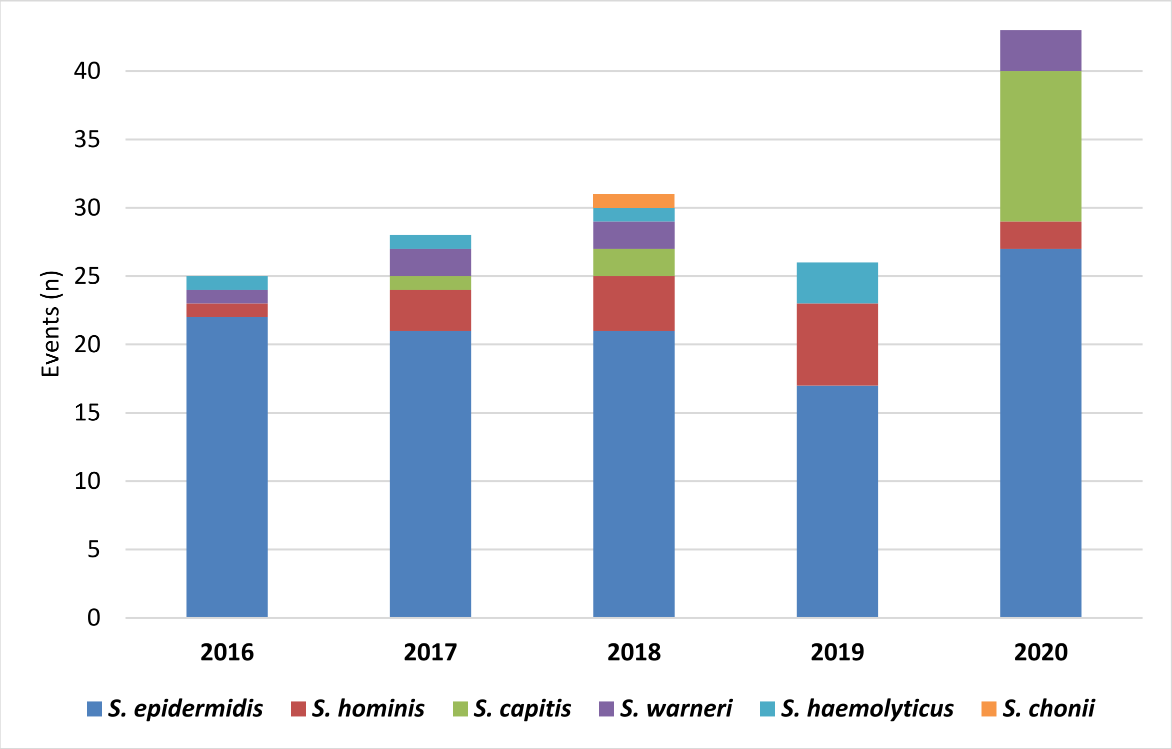

Figure S1: Distribution of CoNS events and species by year

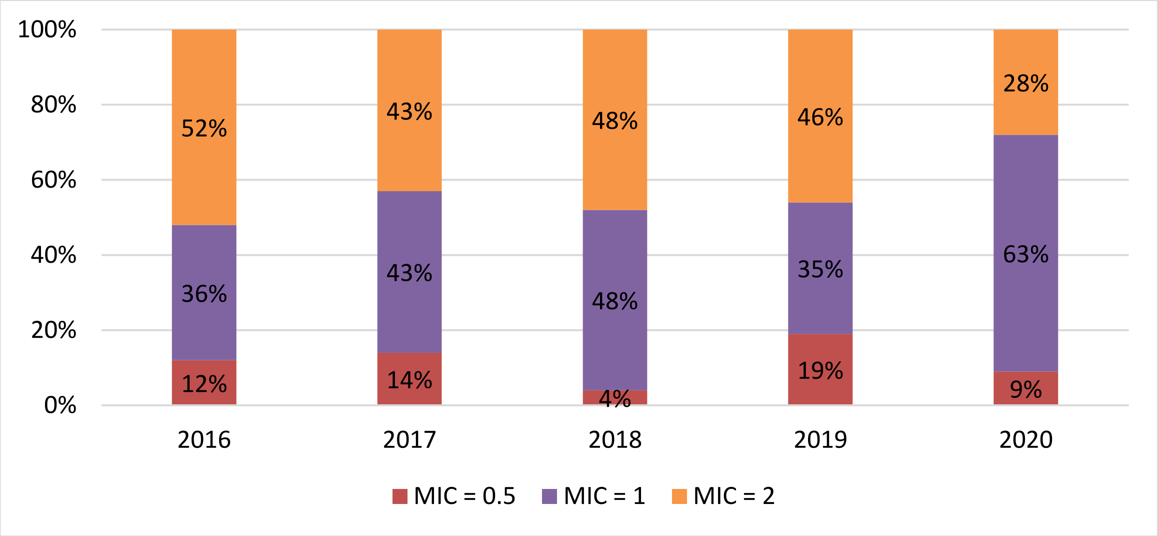

Figure S2: Distribution of MIC for vancomycin in CoNS isolates by year

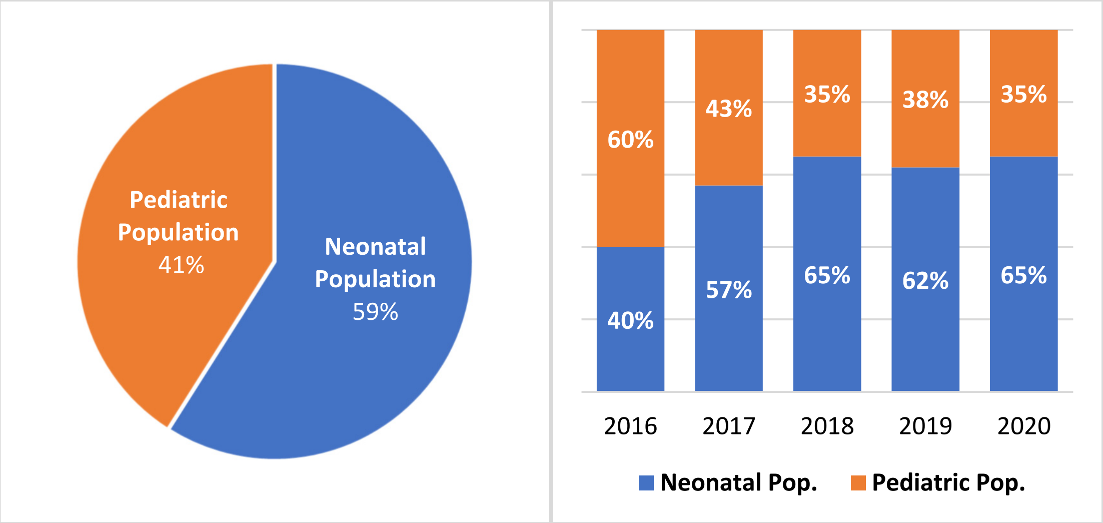

Figure S3: Events distribution in neonatal and pediatric population (total and by single year).

Table S1: Clinical and microbiological characteristics of study events in neonatal population

Features	n (%)
**Events**	90
**Patients**	82
**Female sex**	32 (39)
Reason for NICU admission	
Prematurity	47 (52)
Congenital heart disease	22 (24)
Other congenital gastrointestinal or laryngotracheal anomaly	16 (18)
Hypoxic ischemic encephalopathy	5 (6)
**Presence of CVCs**	84 (94)
**ECMO**	0 (0)
**Dialysis**	0 (0)
**Re-infections at 15 days after therapy stop**	0 (0)
**Mortality at 30 days after infectious event**	9 (10)
Etiology	
S. epidermidis	66 (73)
S. capitis	10 (11)
S. warneri	6 (7)
S. hominis	5 (6)
S. haemolyticus	2 (2)
S. cohnii	1 (1)
Vancomycin MIC of isolates	
0,5	7 (8)
1	45 (50)
2	38 (42)

Table S2: Clinical and microbiological characteristics of pediatric population

Features	n (%)
**Events**	63
**Patients**	58
**Male sex**	33 (57)
Reason for NICU admission	
Cardiac surgery	18 (29)
Thoracic and/or abdominal surgery	16 (25)
Neurological disorder	16 (25)
Hemato-Oncologic disorder	6 (10)
Prematurity	7 (11)
**Presence of CVCs**	61 (97)
**ECMO**	0 (0)
**Dialysis**	0 (0)
**Re-infections at 15 days after therapy stop**	1 (1,6)
**Mortality at 30 days after infectious event**	1 (1,6)
Etiology	

S. epidermidis	42 (67)
S. hominis	11 (17)
S. haemolyticus	4 (6)
S. capitis	4 (6)
S. warneri	2 (3)
Vancomycin MIC of isolates	
0,5	10 (16)
1	27 (43)
2	26 (41)

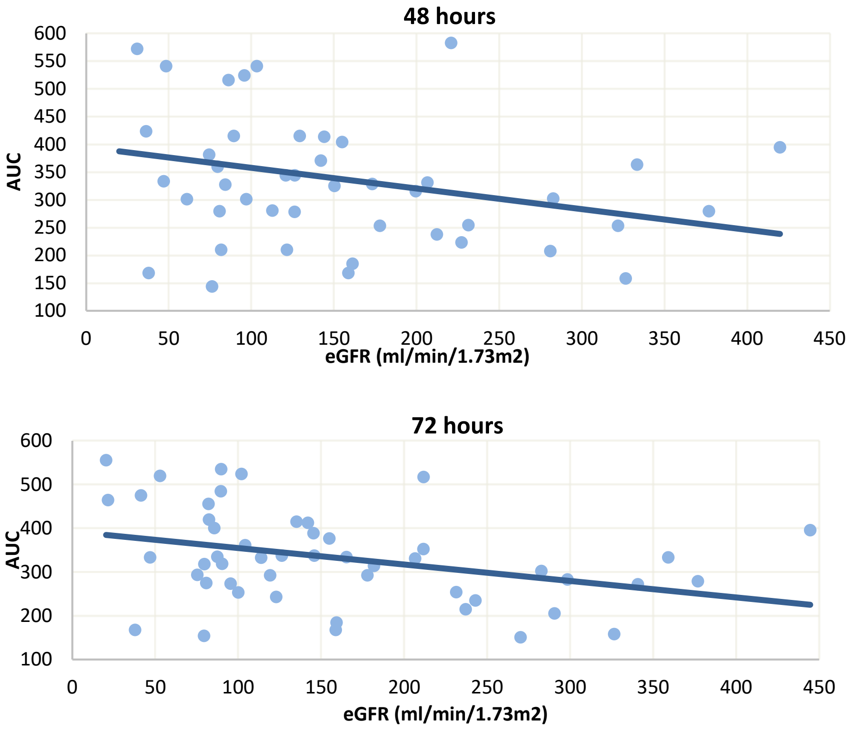

Figure S4 panel A - B: Correlation between AUC and eGFR median values at 48 and 72h of therapy in pediatric population

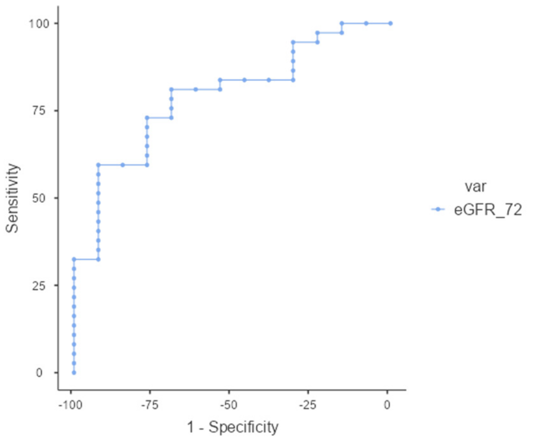

Figure S5: ROC curve of eGFR value at 72h
